# MRI of a painful carpal boss: variations at the extensor carpi radialis brevis insertion and imaging findings in regional traumatic and overuse injuries

**DOI:** 10.1007/s00256-018-3136-9

**Published:** 2019-01-12

**Authors:** Mika T. Nevalainen, Johannes B. Roedl, William B. Morrison, Adam C. Zoga

**Affiliations:** 1 Division of Musculoskeletal Imaging and Intervention, Department of Radiology, Thomas Jefferson University Hospitals, Sidney Kimmel Medical College at Thomas Jefferson University, 132 South 10th Street, Philadelphia, PA 19107 USA; 2 Department of Diagnostic Radiology, Oulu University Hospital, PO Box 50, 90029 Oulu, Finland; 3 Medical Research Center Oulu, University of Oulu, PO Box 8000, Oulu, Finland; 4 Research Unit of Medical Imaging, Physics and Technology, Faculty of Medicine, University of Oulu, PO Box 5000, 90014 Oulu, Finland

**Keywords:** Bone marrow edema, Carpal boss, Extensor carpi radialis brevis, Magnetic resonance imaging, Os styloideum

## Abstract

**Objective:**

To report patterns of MRI findings involving carpal boss and extensor carpi radialis brevis (ECRB) tendon insertion in individuals with overuse-related or post-traumatic wrist pain.

**Materials and Methods:**

Eighty-four MRI cases with carpal bossing between December 2006 and June 2015 were analyzed by two fellowship-trained musculoskeletal radiologists. The following MRI findings were reviewed: type of carpal bossing (bony prominence, partial coalition, os styloideum), insertion of ECRB tendon (to the 3rd metacarpal, to carpal boss or to both), bone marrow edema (BME), insertion site, and tenosynovitis/tendinosis of ECRB tendon. Clinical information on wrist pain was available on 68 patients.

**Results:**

Fused carpal bossing was detected in 21%, partial coalition in 35%, and os styloideum in 44% of the cases. Regional BME was observed in 64% of the cases. When BME specifically at the carpal boss was assessed, 78% of stable and 50% of unstable bosses showed BME (*p* = 0.035). ECRB tendon inserted on a carpal boss in 20%, on the 3rd metacarpal bone in 35%, and on both sites in 45% of the cases. As BME at the carpal boss was assessed, BME was detected at the respective insertion sites in 71%, 35%, and 66% of the cases (*p* = 0.015). Dorsal wrist pain was associated with BME as 75% of the patients had regional BME in the vicinity of the carpal boss (*p* = 0.006).

**Conclusion:**

A spectrum ranging from complete fusion of a boss to an entirely unfused os styloideum exists with a variable ECRB insertional anatomy. BME at the carpal boss is a consistent MRI finding.

## Introduction

Carpal boss is defined as a bony protuberance on the dorsal base of the 3rd metacarpal bone on the quadrangular joint formed by the 2nd and 3rd metacarpals, trapezoid, and capitate. However, the exact definition of the carpal boss remains elusive; to date, no specific measurements exist to define what size such a protuberance meets criteria to be termed a carpal boss. Moreover, the prevalence of the carpal boss is also loosely characterized: in an old study from the 1950s applying hand radiographs Bassoe and Bassoe reported a 1–4% occurrence [1]. Nonetheless, fairly recent cadaveric studies suggest a higher prevalence of 18–19% [2, 3]. The etiology of carpal boss is uncertain and a multitude of explaining theories exist, including congenital, degenerative, overuse, and traumatic etiology [4–8]. A non-united osseous fragment at the dorsal, radial aspect of the 3rd metacarpal base is termed os styloideum, whereas an osseous prominence without fragmentation fits into the more inclusive term carpal boss. Occasionally, the presence of carpal boss can lead to dorsal wrist pain and hand dysfunction, requiring medical care. Interestingly, some studies suggest that the symptomatic carpal boss might be more likely to be on the dominant hand of an individual in their early 30s [3, 4, 6]. In the diagnosis of a symptomatic carpal boss, the magnetic resonance imaging (MRI) plays an essential role [8]; nevertheless, only a few case series on the MRI features of carpal boss exist in the literature [9, 10]. Moreover, the clinical syndrome of a symptomatic carpal boss has been reported and described with an emphasis on osseous anatomy [9, 11]. Regional soft-tissue anatomy, however, is complex and the extensor carpi radialis brevis (ECRB) tendon generally inserts on the base of the 3rd metacarpal, in close proximity to a carpal boss. Anecdotally, we noted several cases of symptomatic carpal boss that had clinical and MRI findings indicating stress or avulsive injury at the ECRB insertion [10]. Along with osseous and soft-tissue edema, there were varying degrees of osseous fragmentation and varying patterns of ECRB insertion. Thus, we hypothesized that osseous and soft-tissue anatomical variations at and around a carpal boss might contribute to pain syndromes at the dorsum of the wrist, and that recognition of these variations might contribute to improved treatment planning. We set out to systematically categorize anatomical variations at the ECRB insertion and the carpal boss, and to describe the patterns of MRI findings associated with the painful carpal boss, including types of os styloideum and the relationship of the ECRB tendon insertion with osseous anatomy.

## Materials and methods

### Subjects

Institutional review board approval was obtained and the requirement for informed consent was waived. A retrospective report review of 3,032 wrist MRIs interpreted at the authors’ institution by subspecialty musculoskeletal radiologists was performed, searching the PACS database with the keywords “carpal boss” and “os styloideum,” and clinical indications were searched for pain at the dorsum of the ipsilateral wrist. MRI studies with confounding pathological ulnar condition, degenerative or inflammatory arthropathy of the carpus, and with clear sources of pain away from the carpal boss region were excluded. Any patients with recent radiographs positive for fracture were also excluded. Accordingly, the search yielded 84 cases with confirmed carpal bossing between December 2006 and June 2015. A pre-MRI questionnaire filled by the patient was assessed to collect information on the age, gender, location of the wrist pain (dorsal or volar), hand dominance, athletic endeavors, and recent wrist trauma or overuse history. Clinical information on wrist pain was available on 68 patients (48 reporting dorsal and 20 volar pain). Moreover, 46 subjects reported a previous wrist trauma and 39 subjects wrist overuse (one patientreported both). The side of hand dominance was available in 64 patients. Eight subjects also had a CT examination of the same ipsilateral wrist within 6 weeks of the MRI.

### MRI, image analysis, and statistics

All wrist MRI was performed on 1.5-Tesla MRI scanners (Optima or Extreme; General Electric Medical Systems, Milwaukee, WI, USA) with dedicated coils and routine protocols in three planes including coronal T1-weighted, proton-density fast spin echo (FSE) fat-suppressed and 2D gradient echo sequences, sagittal T2-weighted FSE, and short tau inversion recovery (STIR) or proton-density FSE fat-suppressed sequences, and an axial T1-weighted and proton density or T2-weighted FSE fat-suppressed sequence. MRI slice thickness was 3 mm in general, except for the gradient echo sequences, where it was 1 mm. CT examinations were performed with helical acquisitions at a 0.63-mm slice thickness, and 2D coronal, sagittal, and axial reformats, in addition to 3D reformats were constructed on a workstation.

Bone marrow edema (BME) was defined as hyperintense signal on fluid-sensitive, fat-suppressed sequences at any part of the carpal boss. The type of carpal bossing was classified as a fully fused bony prominence at the 3rd metacarpal base, partial fusion of the osseous prominence, or an entirely separate/fragmented ossicle (os styloideum). A stable boss was defined as a fully fused prominence at the 3rd metacarpal base, and an unstable boss as a partial fusion or an entirely separate os styloideum. The insertion of the ECRB tendon was classified to the 3rd metacarpal, to the carpal boss/os styloideum, or to both an os styloideum or partially fused carpal boss, and the 3rd metacarpal base (combined insertion). Moreover, the BME at the carpal boss, at the 2nd or 3rd metacarpal and at the adjacent carpal bones, and possible tenosynovitis or tendinosis of ECRB tendon were recorded. Two fellowship-trained musculoskeletal radiologists, blinded to the clinical data, reviewed the MR images of the 84 cases in consensus.

Associations between different parameters were evaluated using the Chi-squared test and *p* < 0.05 was considered statistically significant. Statistical software (version 24.0; SPSS, Chicago, IL, USA) was used for the analysis.

## Results

**Fig. 1:**
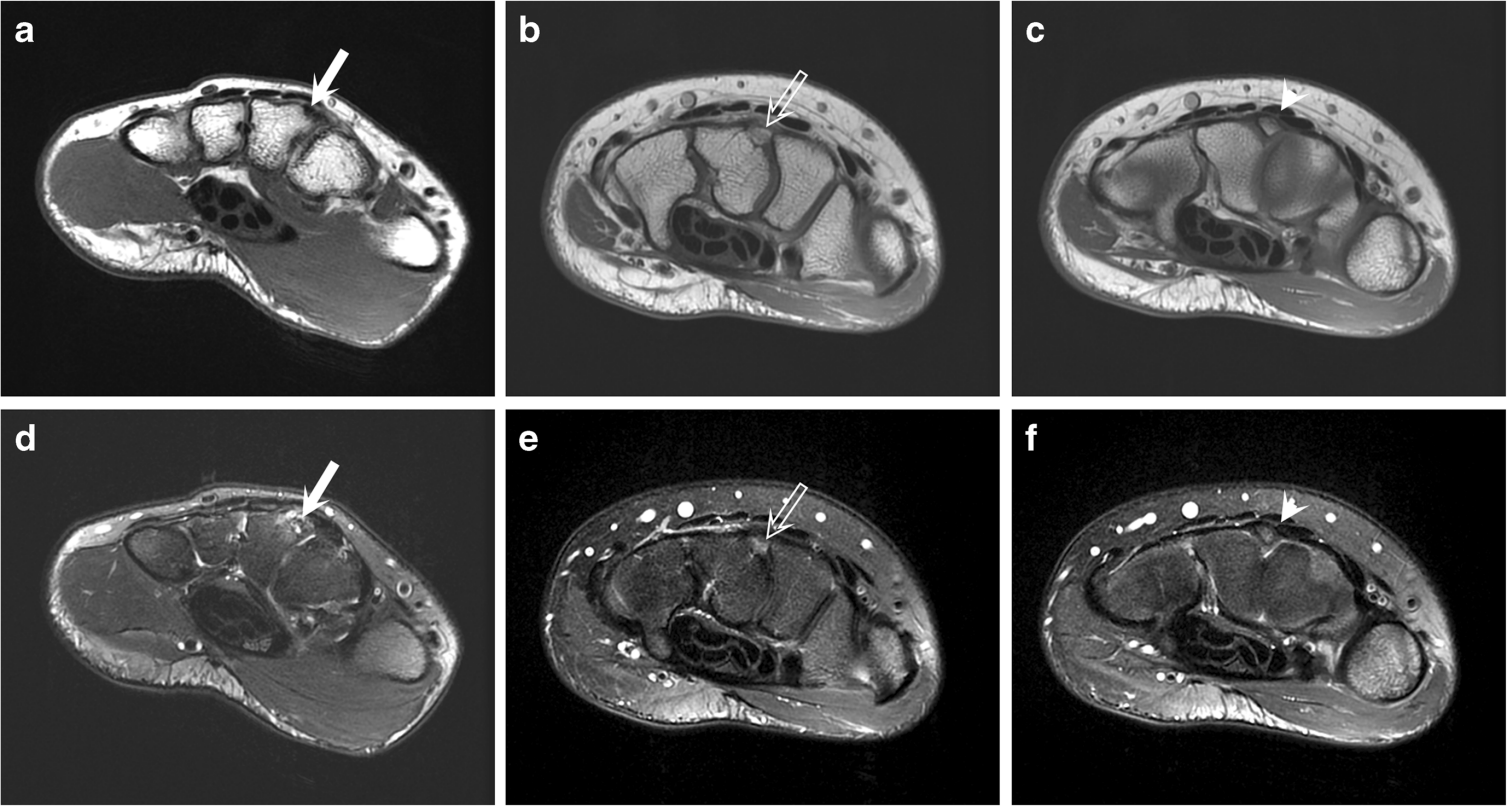
**a–c** T1-weighted and** d–f** T2-weighted fat-saturated axial MR images of the wrist depicting a fused carpal boss (**a**, **d**), a partial coalition (**b**, **e**), and an os styloideum (**c**, **f**). A 46-year-old man with an osseous prominence, i.e., fused carpal boss (*white arrow*) on the base of the 3rd metacarpal with degenerative changes (**a**, **d**). A 21-year-old woman showing a partial coalition of the os styloideum with mild bone marrow edema (*open arrow*; **b**, **e**). A 24-year-old man demonstrating a characteristic unfused os styloideum (*arrowhead*; **c**, **f**)

The mean subject age was 38 years (range 12–69 years), and 63% were males. In total, 84 cases with carpal bossing were identified at MRI: fully fused carpal bossing was logged in 21% (18 out of 84), partial coalitions in 35% (29 out of 84), and an non-united os styloideum in 44% of subjects (37 out of 84; Fig. 1). Overall, regional BME was a common finding, observed in 64% (54 out of 84) of the cases. BME was isolated to the boss or the os styloideum in 56% of cases (47 out of 84), at the base of the 2nd metacarpal in 12% of cases (10 out of 84), at the base of the 3rd metacarpal in 38% (32 out of 84) of cases, and at the adjacent carpal bones in 6% of cases (5 out of 84). Clear fractures involving the fused or partially fused carpal boss on the dorsum of the base of the 3rd metacarpal were identified in 4 cases (3 fused, 1 partially fused). Within this small subset, all 4 reported an activity involving repeated forced wrist extension (3 ice hockey, 1 field hockey). The ECRB tendon insertion was anatomically variable. The ECRB inserted entirely on the 3rd metacarpal base in 35% (29 out of 84), entirely on an os styloideum or partially fused carpal boss fragment in 20% (17 out of 84), and on both the os styloideum or partially fused fragment and the 3rd metacarpal base (combined insertion) in 45% (38 out of 84). Distal ECRB tenosynovitis/tendinosis was observed in 15% (13 out of 84) of the cases. In 63% of the subjects (40 out of 64), the dominant hand was imaged.

With regard to different carpal boss types, regional BME was present in each anatomical group, including 83% (15 out of 18) with a fused bony prominence at the 3rd metacarpal base, 55% (16 out of 29) with partial coalition, and 62% (23 out of 37) with an os styloideum. Although regional BME was more associated with a stable than an unstable boss (83% [15 out of 18] vs 59% [39 out of 66]), this finding was statistically insignificant (*p* = 0.057). However, when BME isolated to the carpal boss was assessed, it was present in 78% of stable (14 out of 18) and 50% of unstable bosses (33 out of 66; *p* = 0.035). Regional BME was associated with younger subject age, with a mean age of subjects with regional BME of 34 years and a mean age of subjects without BME of 47 years (*p* < 0.001). Correlating BME with the type of ECRB insertion, regional BME at the vicinity of the carpal boss was seen in 55% (16 out of 29) with a full 3rd metacarpal base insertion, in 71% (12 out of 17) with the ECRB insertion on an os styloideum or partially fused carpal boss, and in 68% (26 out of 38) with the combined insertion site. There was no statistically significant difference in frequency of BME between the different ECRB insertion groups (*p* = 0.443). On further analysis, where BME was isolated to the carpal boss region, it was present at the respective insertion sites in 35% (10 out of 29), 71% (12 out of 17), and 66% (25 out of 38) of the subjects (*p* = 0.015). The variance of the ECRB insertion site was not associated with ECRB tenosynovitis/tendinosis (*p* = 0.383).

**Fig. 2:**
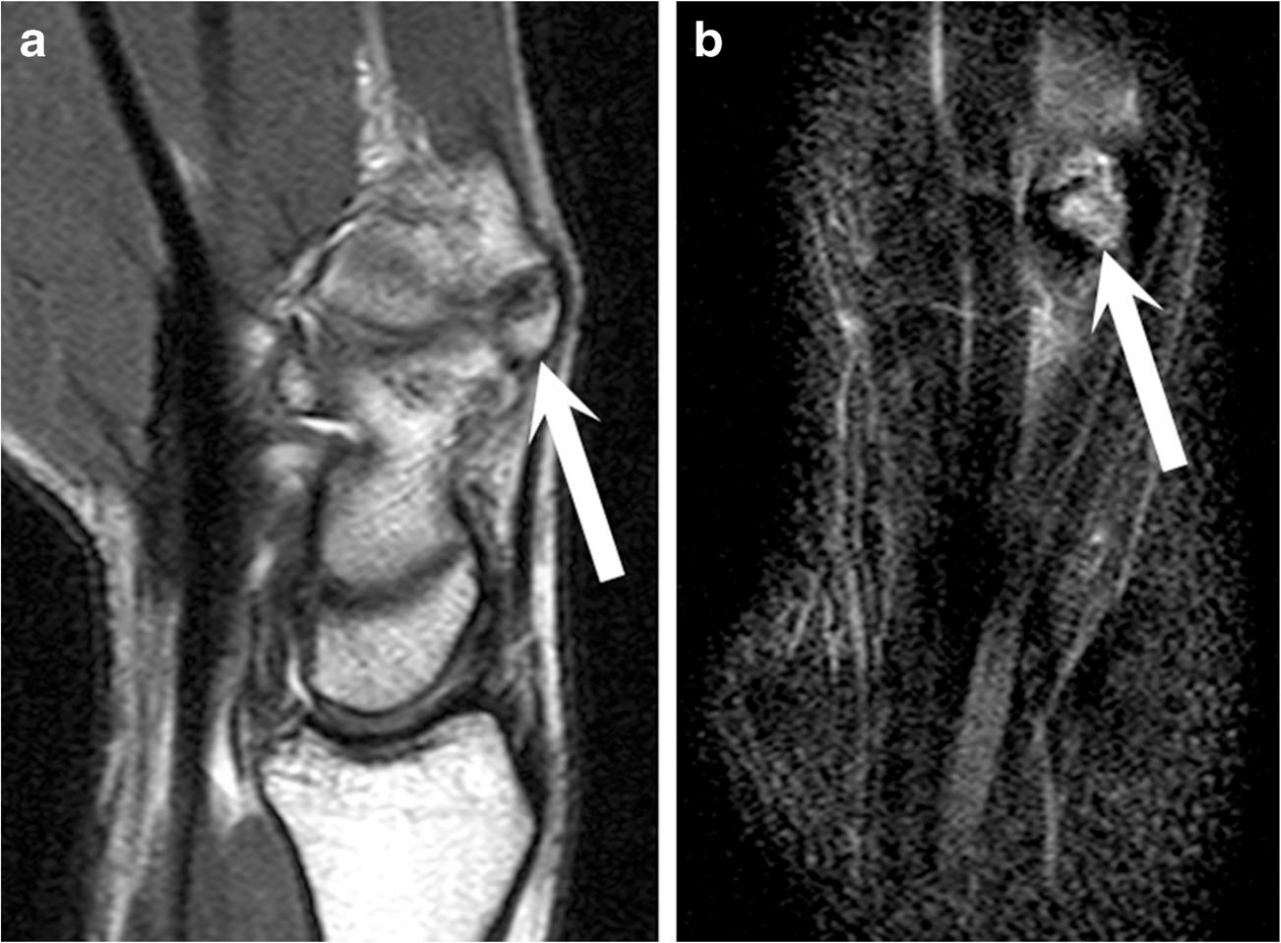
A 23-year-old professional male ice hockey player with persistent dorsal wrist pain.** a** T1-weighted and** b** T2-weighted fat-saturated sagittal MR images demonstrate an unfused carpal boss, i.e., os styloideum with marked bone marrow edema (*arrow*)

**Fig. 3:**
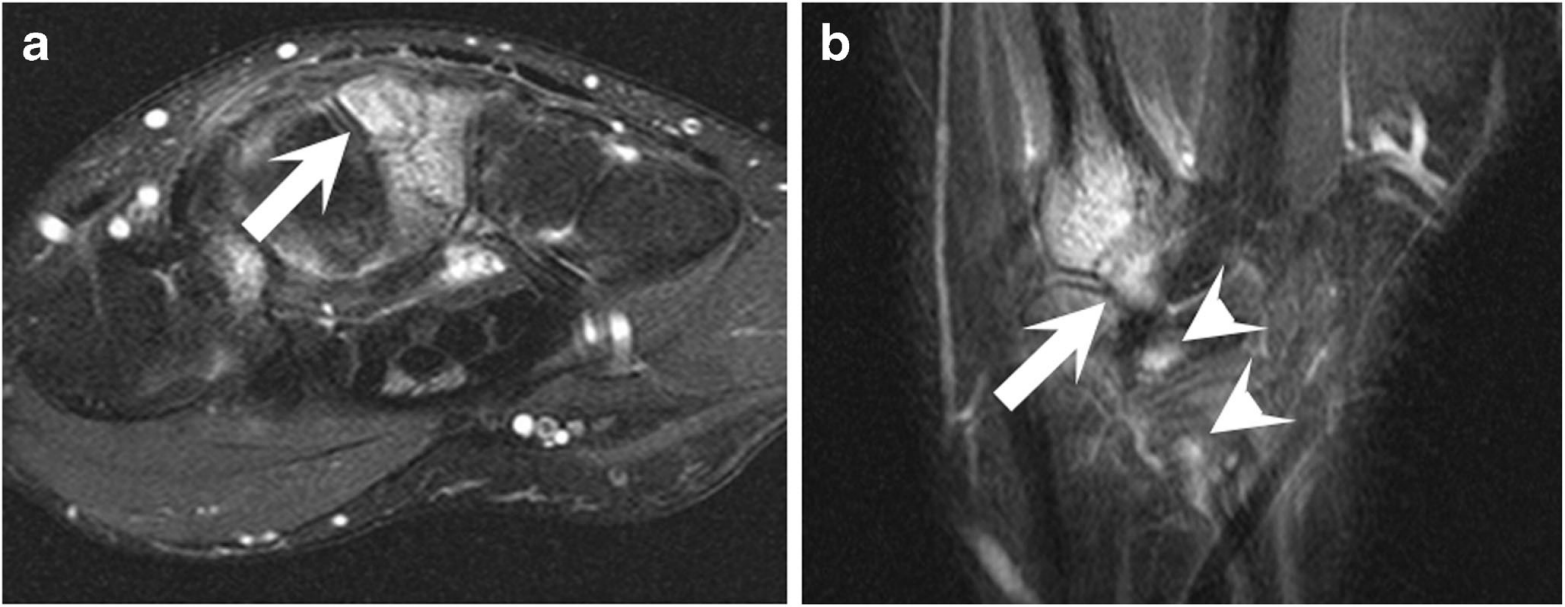
**a** Axial proton-density-weighted fat-suppressed image and** b** a coronal proton-density-weighted fat-suppressed image from a wrist MRI performed on a 38-year-old man after hyperflexion trauma in a fall shows a fracture at a partially fused carpal boss, with extensive bone marrow edema (*white arrows*) and extensive tendinous edema in the distal extensor carpi radialis brevis tendon (*white arrowheads*)

Ultimately, a painful carpal boss or injury to the carpal boss was clinically suspected before MRI in 48 subjects. With analysis of this subgroup, we observed that 75% of the patients (36 out of 48) had focal BME at the carpal boss (*p* = 0.006; Figs. 2, 3) Further, 19% (9 out of 48) had a fused carpal boss, 46% (22 out of 48) had partial coalition, and 35% (17 out of 48) had os styloideum. There was no statistical significance between anatomical instability of the carpal boss and a documented clinically painful carpal boss, but out of the 48 painful carpal bosses, 19% (9 out of 48) were stable and the remaining 81% (39 out of 48) were unstable (*p* = 0.150). Accordingly, 29% (14 out of 48) had an ECRB insertion on the 3rd metacarpal base, 25% (12 out of 48) had an ECRB insertion on a partially fused or unfused osseous fragment, and 46% (22 out of 48) had a combined insertion. There was no statistically significant difference in the association of BME with ECRB insertion type in this subset (*p* = 0.386).

## Discussion

Throughout the body, musculoskeletal syndromes involving pain at sites of ossicles, sesamoids, or apophyses almost always involve tendinous and capsular attachments. Musculoskeletal anatomy at the dorsum of the carpal–metacarpal region is intricate and somewhat complex, with osseous, articular, and soft-tissue variations. Although the painful carpal boss is a well-published clinical syndrome, little work has examined the relationship between biomechanical avulsive forces at the ECRB insertion and symptoms/anatomical variations. Mespreuve et al. reviewed 57 subjects with carpal boss who underwent radiography and MRI, and reported that 73% of the patients had fused carpal bosses, 4% had partial coalitions, and the remaining 23% had an os styloideum [9]. Goiney et al. reviewed 129 wrist CTs of the general population with no suspicion of dorsal wrist pain or carpal boss, and reported a very high proportion of subjects (82%) showing a dorsal protuberance, 7% showing an os styloideum, and 11% showing no carpal boss [11].

We used MRI to examine osseous variations that fall within the anatomical spectrum of a carpal boss, in addition to the soft-tissue variations at the ECRB tendon insertion, and their relationship. We focused on a subject group with regional symptoms, many of whom were sent to MRI with a clinical diagnosis of a painful carpal boss. To our knowledge, this is the first study to detail both osseous and soft-tissue findings at MRI along with anatomical variations in the clinical setting of a painful carpal boss. We ultimately found a great deal of anatomical variation that almost certainly had an impact on clinical symptoms. As for the osseous anatomy, we observed a fused carpal boss in 21%, ossific fragments that are partially fused to the dorsum of the base of the 3rd metacarpal (partial coalitions) in 35%, and isolated, fully unfused os styloideum in 44% of subjects. As for ECRB tendon insertion, we found a full 3rd metacarpal bone insertion in 35%, an insertion on an os styloideum or partially fused carpal boss in 20%, and a combined insertion, with tendon fibers attaching to both the 3rd metacarpal base and others attaching to an os styloideum, in 45% of subjects. This anatomy can be considered somewhat analogous to the posterior tibial tendon insertion on or over type 1, type 2, and type 3 os naviculare at the medial navicular, another musculoskeletal region rife with variation and prone to painful syndromes [12].

In our subject group, regional BME near carpal boss reflecting previous trauma or overuse of the dorsal carpus was present in 64% of the cases (54 out of 84). This is increased compared with the 28% (16 out of 57) reported in the Mespreuve group in 2017 [9]. This likely reflects subject selection criteria, as all of our subjects reported dorsal wrist pain and previous trauma or history of wrist overuse. A similarly high incidence of regional BME has been shown by a small study by Greditzer et al. on National Hockey League players, reporting that 91% (10 out of 11) of the players with carpal boss show regional BME [10]. In our group, regional BME was more common with younger patients. This may reflect a more focused clinical algorithm directing younger patients to MRI, or it may simply reflect a subset of ice hockey players that was sewn into our study cohort.

Overall, BME was more frequently observed in subjects with carpal bosses fully fused to the 3rd metacarpal base, but in the subset with a clinical diagnosis of painful carpal boss, BME was more common, with partially fused or unfused ossifications. Although not statistically significant, it stands to reason that osseous instability often plays some role in the clinically symptomatic carpal boss. We propose that one scenario involves repetitive micro-avulsive extension stress at the ECRB insertion on or over an unstable carpal boss. In other cases, however, there were discrete traumas to the dorsum of the hand in the vicinity of the carpal boss. Another clinical scenario is likely pain with extension after soft-tissue and/or osseous edema infiltrates the region following a trauma. This is analogous to anterior knee pain during extension after a direct contusion to the patella.

The variability of the insertion site of the ECRB tendon showed no significant correlation with regional BME in our cohort, as 55% with the metacarpal insertion, 71% with the os styloideum/carpal boss insertion, and 68% with the combined insertion site showed BME (*p* = 0.443). However, this anatomical variation should be detailed on imaging reports, as it may have an impact on intervention, whether surgical or percutaneous. Although not statistically significant, in the clinical painful carpal boss subset, there was more BME in subjects who showed an ECRB insertion on an os styloideum or partially fused ossific fragment than in those with the combined insertion and the full insertion on the base of the 3rd metacarpal (*p* = 0.015). BME and pain may be more likely if the ERCB inserts directly on an unstable osseous fragment, and these patients may be the best candidates for surgical resection of the ossific fragment. Furthermore, 75% of the symptomatic subjects with a clinical diagnosis of painful carpal boss showed regional BME, and of these, BME was more frequent with an unfused ossific fragment. Intuitively, it appeared that the instability of the carpal boss would also be associated with BME (81% vs 19%). To date, only 4 case reports exist depicting subjects with dorsal wrist associated with regional BME on MRI [13–15]. Although the literature on painful carpal boss is fairly scarce, it can be concluded that BME shows a strong association with painful carpal boss.

Computed tomography does play a role in imaging the osseous hand and wrist after trauma when radiographs are normal or equivocal, but it lacks sensitivity for BME and specificity for tendinous detail. In our series, CT was ordered sparingly when there were clinical or MRI findings concerning for fracture. In the three cases in which carpal boss fracture was identified at MRI and a CT was ordered, CT confirmed all three fractures. In other patients who underwent CT, non-united or partially fused os styloideum was confirmed.

There are limitations in this study. We have no clinical follow-up, as this retrospective review is focused on clinical information and findings at the time of the MRI. We have no surgical correlation to prove the anatomical variations observed, although imaging quality is strong. Clinical information is limited to the imaging orders and patient questionnaires, without direct interaction with managing clinicians.

In conclusion, overuse and traumatic injury involving a carpal boss is not uncommon, and unenhanced MRI is an optimal modality for identifying BME, soft-tissue injury, and anatomical variations. BME at the carpal boss or at an os styloideum is a consistent MRI finding in patients with dorsal wrist and no other MRI abnormality. There is great anatomical variation, and a spectrum of osseous anatomy exists, ranging from complete fusion of a boss to the 3rd metacarpal base to an entirely unfused os styloideum. ECRB insertional anatomy is also variable, ranging from insertion on a fixed boss to a shared insertion between an os styloideum and the metacarpal base. This injury pattern at MRI can reflect repetitive extension trauma at the wrist or a single dorsal hand/wrist trauma. Musculoskeletal imagers should recognize this pattern of injury and should delineate the variable anatomy at both the carpal boss/os styloideum and at the ECRB insertion. With MRI guidance, treatment algorithms can be tailored to the individual anatomy and injury.
